# Supplementary material for: Biogeography of the Caribbean Cyrtognatha spiders
Source: Sci Rep. 2019 Jan 23;9:397. doi: 10.1038/s41598-018-36590-y (PMC6344596; doi:10.1038/s41598-018-36590-y)
Supplement: Supplementary file 1 — Supplementary material [file 41598_2018_36590_MOESM1_ESM.docx]

**Biogeography of the Caribbean *Cyrtognatha* spiders**

*Klemen Čandek^1,6^, Ingi Agnarsson^2,4^, Greta J. Binford^3^, Matjaž Kuntner^1,4,5,6^

**Supplementary Figure S1**

Unconstrained, single gene (COI), approach to the all-terminal Bayesian phylogeny.


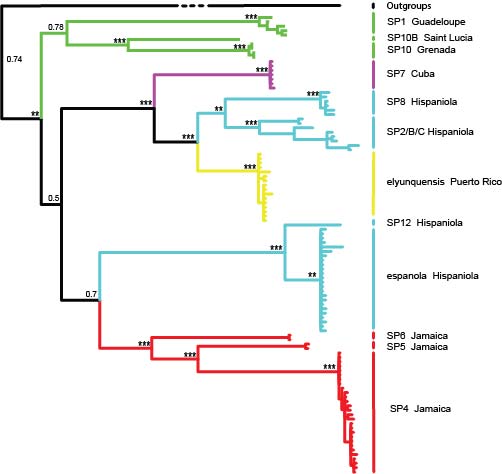


**Supplementary Figure S2**

All-terminal (COI) Bayesian phylogeny with constrained basal Caribbean *Cyrtognatha* node. The constraint was made based on the two gene species level phylogeny due to known software difficulties on root placement in phylogenies using only a single mitochondrial gene. This Supplementary Figure S2 is the same as Figure 1 but has outgroups and specimen details displayed. HSP = Hispaniola; LA = Lesser Antilles; PR = Puerto Rico; JAM = Jamaica


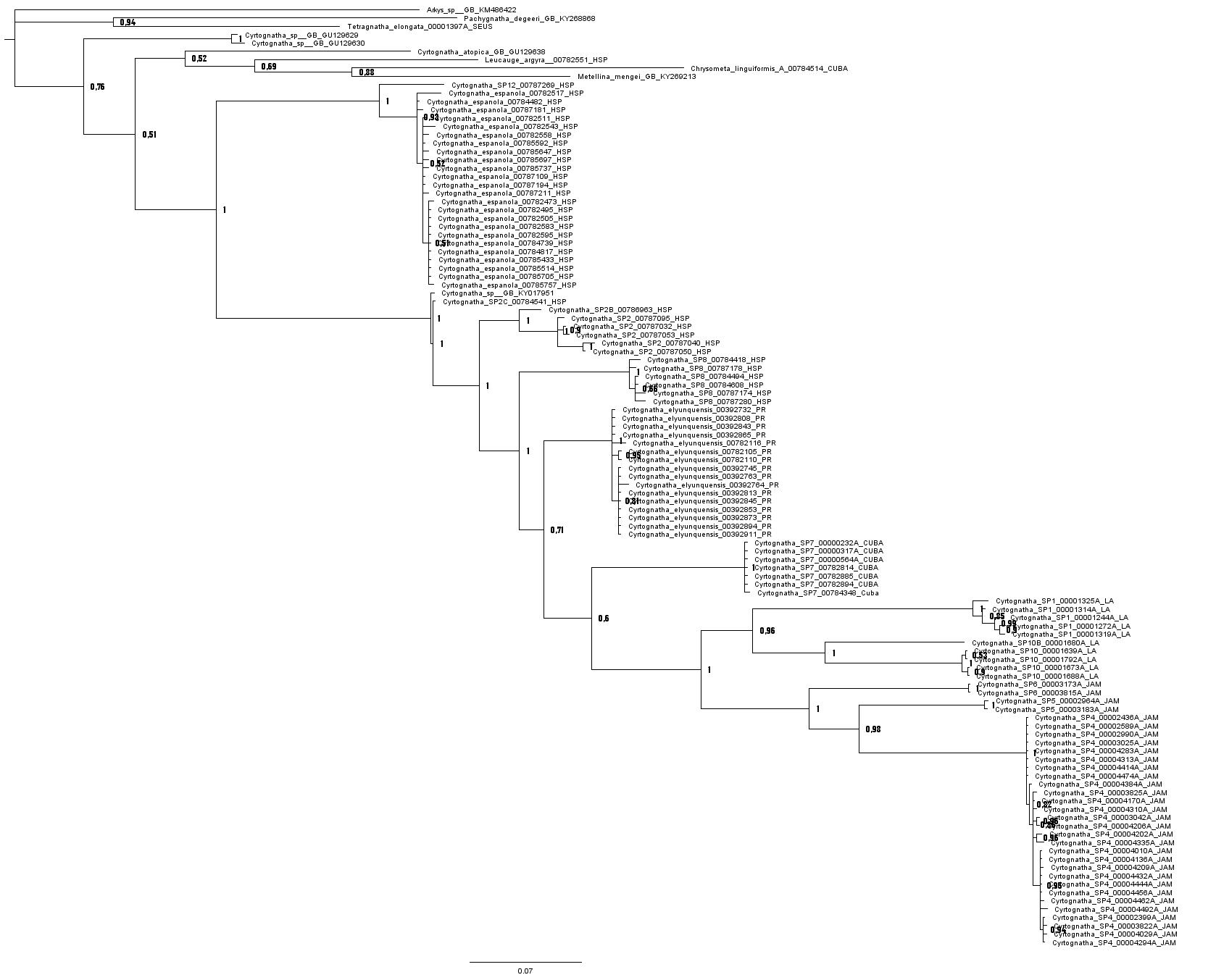


**Supplementary Figure S3**

Two gene (COI and 28S) species level Bayesian phylogeny with specimen details displayed. HSP = Hispaniola; LA = Lesser Antilles; PR = Puerto Rico; JAM = Jamaica


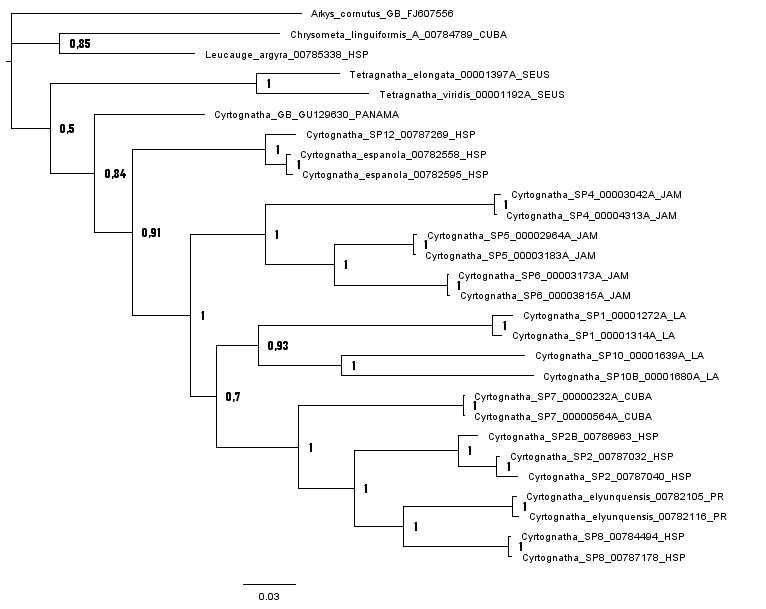


**Supplementary Figure S4**

BEAST chronogram using COI mutation rate for time calibration. Specimen details are displayed. HSP = Hispaniola; LA = Lesser Antilles; PR = Puerto Rico; JAM = Jamaica


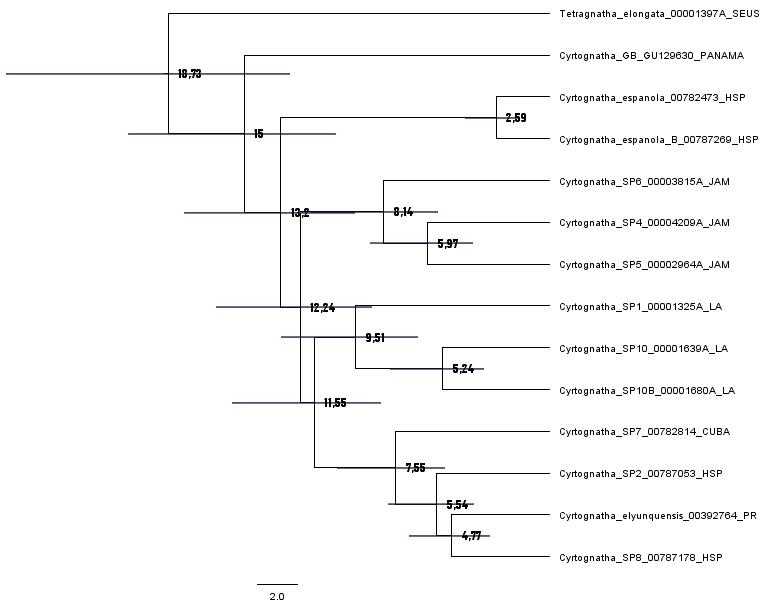


**Supplementary Figure S5**

BEAST chronogram using COI mutation rate and *Cyrtognatha weitschati* fossil for time calibration. Specimen details are displayed. HSP = Hispaniola; LA = Lesser Antilles; PR = Puerto Rico; JAM = Jamaica


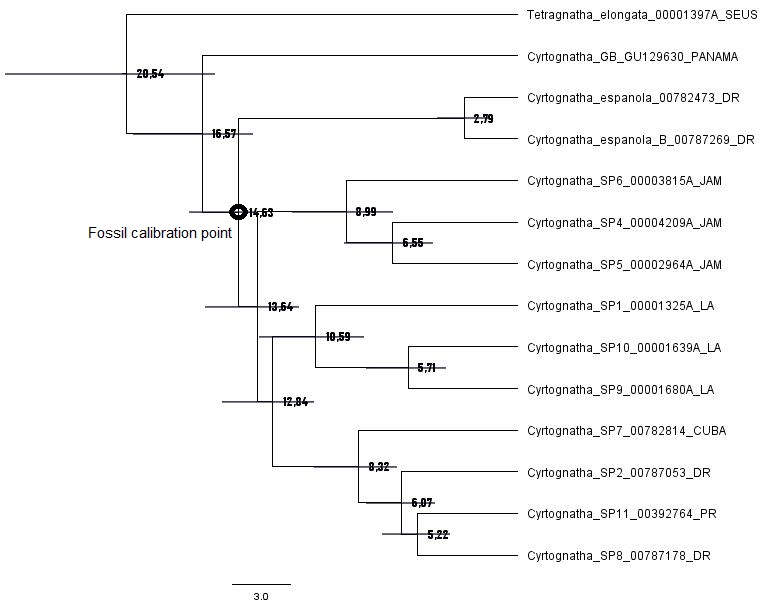


**Supplementary Figure S6**

Ancestral area estimation of *Cyrtognatha* with BioGeoBEARS using DIVALIKE (max_range_size = 2). This is an alternative to the ancestral area estimation from Figure 4. In this analysis we did not use the founder event parameter (+J) due to concerns recently raised by Ree and Sanmartin (2018) about the statistical concept of +J parameter.

However, when we eliminated the +J parameter, the results from DIVALIKE ancestral area estimation produced controversial results. The results suggested purely vicariant origins of all *Cyrtognatha* subclades which clearly does not fit the known geologic history of the Caribbean. A clear example are de novo formed volcanic Lesser Antilles and reemergence of Jamaica, both around 12 - 10 MYA. Therefore, the ancestral range of Hispaniola + Lesser Antilles and Hispaniola + Jamaica is not logical. Moreover, the timing of the remaining vicariant processes (events following the nodes with ancestral range of Panama + Hispaniola, Hispaniola + Cuba and Hispaniola + Puerto Rico) also do not fit the Caribbean geologic history. Therefore, within the constraints of our study, we find the models using +J parameter more appropriate.


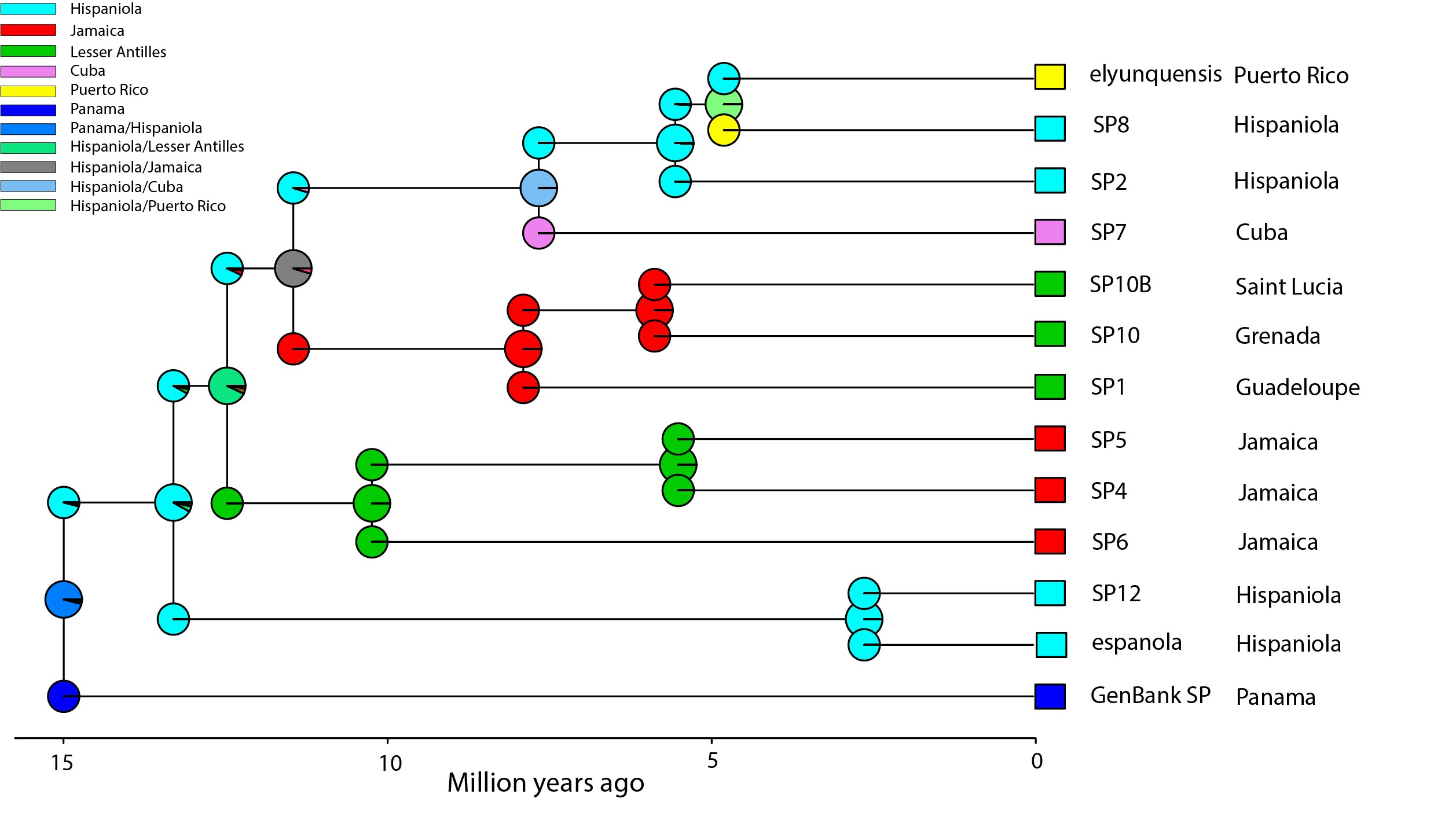


**Supplementary Note S1**

*Multi-rate Poisson tree process (mPTP) species delimitation results:*

mCommand: ../bin/mptp mptp --ml --multi --outgroup Tetragnatha_elongata_00001397A_SEUS --outgroup_crop --tree_file ../uploads/h1r5ushkj2d574npgdjgliok41.newick --output_file ../uploads/h1r5ushkj2d574npgdjgliok41.1

Number of edges greater than minimum branch length: 73 / 210

Null-model score: 221.193390

Best score for multi coalescent rate: 221.193390

Number of delimited species: 12

Species 1:

Cyrtognatha_SP4_00004444A_JAM

Cyrtognatha_SP4_00004456A_JAM

Cyrtognatha_SP4_00004432A_JAM

Cyrtognatha_SP4_00004209A_JAM

Cyrtognatha_SP4_00004136A_JAM

Cyrtognatha_SP4_00004010A_JAM

Cyrtognatha_SP4_00004462A_JAM

Cyrtognatha_SP4_00002399A_JAM

Cyrtognatha_SP4_00003822A_JAM

Cyrtognatha_SP4_00004029A_JAM

Cyrtognatha_SP4_00004294A_JAM

Cyrtognatha_SP4_00004492A_JAM

Cyrtognatha_SP4_00004206A_JAM

Cyrtognatha_SP4_00003042A_JAM

Cyrtognatha_SP4_00003825A_JAM

Cyrtognatha_SP4_00004170A_JAM

Cyrtognatha_SP4_00004310A_JAM

Cyrtognatha_SP4_00004202A_JAM

Cyrtognatha_SP4_00004335A_JAM

Cyrtognatha_SP4_00004384A_JAM

Cyrtognatha_SP4_00002436A_JAM

Cyrtognatha_SP4_00002589A_JAM

Cyrtognatha_SP4_00002990A_JAM

Cyrtognatha_SP4_00003025A_JAM

Cyrtognatha_SP4_00004283A_JAM

Cyrtognatha_SP4_00004313A_JAM

Cyrtognatha_SP4_00004414A_JAM

Cyrtognatha_SP4_00004474A_JAM

Species 2:

Cyrtognatha_SP5_00002964A_JAM

Cyrtognatha_SP5_00003183A_JAM

Species 3:

Cyrtognatha_SP6_00003173A_JAM

Cyrtognatha_SP6_00003815A_JAM

Species 4:

Cyrtognatha_SP1_00001272A_LA

Cyrtognatha_SP1_00001319A_LA

Cyrtognatha_SP1_00001244A_LA

Cyrtognatha_SP1_00001314A_LA

Cyrtognatha_SP1_00001325A_LA

Species 5:

Cyrtognatha_SP10B_00001680A_LA

Cyrtognatha_SP10_00001639A_LA

Cyrtognatha_SP10_00001673A_LA

Cyrtognatha_SP10_00001688A_LA

Cyrtognatha_SP10_00001792A_LA

Species 6:

Cyrtognatha_SP7_00782894_CUBA

Cyrtognatha_SP7_00784348_Cuba

Cyrtognatha_SP7_00782885_CUBA

Cyrtognatha_SP7_00782814_CUBA

Cyrtognatha_SP7_00000564A_CUBA

Cyrtognatha_SP7_00000317A_CUBA

Cyrtognatha_SP7_00000232A_CUBA

Species 7:

Cyrtognatha_SP8_00787174_HSP

Cyrtognatha_SP8_00787280_HSP

Cyrtognatha_SP8_00784494_HSP

Cyrtognatha_SP8_00784608_HSP

Cyrtognatha_SP8_00784418_HSP

Cyrtognatha_SP8_00787178_HSP

Species 8:

Cyrtognatha_SP2_00787032_HSP

Cyrtognatha_SP2_00787053_HSP

Cyrtognatha_SP2_00787040_HSP

Cyrtognatha_SP2_00787095_HSP

Cyrtognatha_SP2_00787050_HSP

Cyrtognatha_SP2B_00786963_HSP

Cyrtognatha_sp._GB_KY017951

Cyrtognatha_SP2C_00784541_HSP

Species 9:

Cyrtognatha_elyunquensis_00782116_PR

Cyrtognatha_elyunquensis_00782105_PR

Cyrtognatha_elyunquensis_00782110_PR

Cyrtognatha_elyunquensis_00392843_PR

Cyrtognatha_elyunquensis_00392865_PR

Cyrtognatha_elyunquensis_00392808_PR

Cyrtognatha_elyunquensis_00392732_PR

Cyrtognatha_elyunquensis_00392764_PR

Cyrtognatha_elyunquensis_00392745_PR

Cyrtognatha_elyunquensis_00392763_PR

Cyrtognatha_elyunquensis_00392813_PR

Cyrtognatha_elyunquensis_00392845_PR

Cyrtognatha_elyunquensis_00392853_PR

Cyrtognatha_elyunquensis_00392873_PR

Cyrtognatha_elyunquensis_00392894_PR

Cyrtognatha_elyunquensis_00392911_PR

Species 10:

Cyrtognatha_SP12_00787269_HSP

Species 11:

Cyrtognatha_espanola_00782517_HSP

Cyrtognatha_espanola_00782543_HSP

Cyrtognatha_espanola_00787181_HSP

Cyrtognatha_espanola_00784482_HSP

Cyrtognatha_espanola_00785697_HSP

Cyrtognatha_espanola_00787109_HSP

Cyrtognatha_espanola_00787194_HSP

Cyrtognatha_espanola_00785592_HSP

Cyrtognatha_espanola_00782558_HSP

Cyrtognatha_espanola_00782511_HSP

Cyrtognatha_espanola_00785647_HSP

Cyrtognatha_espanola_00785737_HSP

Cyrtognatha_espanola_00787211_HSP

Cyrtognatha_espanola_00782473_HSP

Cyrtognatha_espanola_00782495_HSP

Cyrtognatha_espanola_00782505_HSP

Cyrtognatha_espanola_00782583_HSP

Cyrtognatha_espanola_00782595_HSP

Cyrtognatha_espanola_00784739_HSP

Cyrtognatha_espanola_00784817_HSP

Cyrtognatha_espanola_00785433_HSP

Cyrtognatha_espanola_00785514_HSP

Cyrtognatha_espanola_00785705_HSP

Cyrtognatha_espanola_00785757_HSP

Species 12:

Cyrtognatha_sp._GB_GU129629

Cyrtognatha_sp._GB_GU129630

*Automatic Barcode Gap Discovery (ABGD) delimitation results:*

Initial Partition with prior maximal distance P=2.60e-02

Distance K80 Kimura MinSlope=5.000000

Group[ 1 ] n: 24 ;id: Cyrtognatha_espanola_00782473_HSP Cyrtognatha_espanola_00782495_HSP Cyrtognatha_espanola_00782505_HSP Cyrtognatha_espanola_00782511_HSP Cyrtognatha_espanola_00782517_HSP Cyrtognatha_espanola_00782543_HSP Cyrtognatha_espanola_00782558_HSP Cyrtognatha_espanola_00782583_HSP Cyrtognatha_espanola_00782595_HSP Cyrtognatha_espanola_00784482_HSP Cyrtognatha_espanola_00784739_HSP Cyrtognatha_espanola_00784817_HSP Cyrtognatha_espanola_00785433_HSP Cyrtognatha_espanola_00785514_HSP Cyrtognatha_espanola_00785592_HSP Cyrtognatha_espanola_00785647_HSP Cyrtognatha_espanola_00785697_HSP Cyrtognatha_espanola_00785705_HSP Cyrtognatha_espanola_00785737_HSP Cyrtognatha_espanola_00785757_HSP Cyrtognatha_espanola_00787109_HSP Cyrtognatha_espanola_00787181_HSP Cyrtognatha_espanola_00787194_HSP Cyrtognatha_espanola_00787211_HSP

Group[ 2 ] n: 1 ;id: Cyrtognatha_SP12_00787269_HSP

Group[ 3 ] n: 2 ;id: Cyrtognatha_sp._GB_GU129629 Cyrtognatha_sp._GB_GU129630

Group[ 4 ] n: 2 ;id: Cyrtognatha_sp._GB_KY017951 Cyrtognatha_SP2C_00784541_HSP

Group[ 5 ] n: 5 ;id: Cyrtognatha_SP1_00001244A_LA Cyrtognatha_SP1_00001272A_LA Cyrtognatha_SP1_00001314A_LA Cyrtognatha_SP1_00001319A_LA Cyrtognatha_SP1_00001325A_LA

Group[ 6 ] n: 4 ;id: Cyrtognatha_SP10_00001639A_LA Cyrtognatha_SP10_00001673A_LA Cyrtognatha_SP10_00001688A_LA Cyrtognatha_SP10_00001792A_LA

Group[ 7 ] n: 16 ;id: Cyrtognatha_elyunquensis_00392732_PR Cyrtognatha_elyunquensis_00392745_PR Cyrtognatha_elyunquensis_00392763_PR Cyrtognatha_elyunquensis_00392764_PR Cyrtognatha_elyunquensis_00392808_PR Cyrtognatha_elyunquensis_00392813_PR Cyrtognatha_elyunquensis_00392843_PR Cyrtognatha_elyunquensis_00392845_PR Cyrtognatha_elyunquensis_00392853_PR Cyrtognatha_elyunquensis_00392865_PR Cyrtognatha_elyunquensis_00392873_PR Cyrtognatha_elyunquensis_00392894_PR Cyrtognatha_elyunquensis_00392911_PR Cyrtognatha_elyunquensis_00782105_PR Cyrtognatha_elyunquensis_00782110_PR Cyrtognatha_elyunquensis_00782116_PR

Group[ 8 ] n: 6 ;id: Cyrtognatha_SP2B_00786963_HSP Cyrtognatha_SP2_00787032_HSP Cyrtognatha_SP2_00787040_HSP Cyrtognatha_SP2_00787050_HSP Cyrtognatha_SP2_00787053_HSP Cyrtognatha_SP2_00787095_HSP

Group[ 9 ] n: 28 ;id: Cyrtognatha_SP4_00002399A_JAM Cyrtognatha_SP4_00002436A_JAM Cyrtognatha_SP4_00002589A_JAM Cyrtognatha_SP4_00002990A_JAM Cyrtognatha_SP4_00003025A_JAM Cyrtognatha_SP4_00003042A_JAM Cyrtognatha_SP4_00003822A_JAM Cyrtognatha_SP4_00003825A_JAM Cyrtognatha_SP4_00004010A_JAM Cyrtognatha_SP4_00004029A_JAM Cyrtognatha_SP4_00004136A_JAM Cyrtognatha_SP4_00004170A_JAM Cyrtognatha_SP4_00004202A_JAM Cyrtognatha_SP4_00004206A_JAM Cyrtognatha_SP4_00004209A_JAM Cyrtognatha_SP4_00004283A_JAM Cyrtognatha_SP4_00004294A_JAM Cyrtognatha_SP4_00004310A_JAM Cyrtognatha_SP4_00004313A_JAM Cyrtognatha_SP4_00004335A_JAM Cyrtognatha_SP4_00004384A_JAM Cyrtognatha_SP4_00004414A_JAM Cyrtognatha_SP4_00004432A_JAM Cyrtognatha_SP4_00004444A_JAM Cyrtognatha_SP4_00004456A_JAM Cyrtognatha_SP4_00004462A_JAM Cyrtognatha_SP4_00004474A_JAM Cyrtognatha_SP4_00004492A_JAM

Group[ 10 ] n: 2 ;id: Cyrtognatha_SP5_00002964A_JAM Cyrtognatha_SP5_00003183A_JAM

Group[ 11 ] n: 2 ;id: Cyrtognatha_SP6_00003173A_JAM Cyrtognatha_SP6_00003815A_JAM

Group[ 12 ] n: 7 ;id: Cyrtognatha_SP7_00000232A_CUBA Cyrtognatha_SP7_00000317A_CUBA Cyrtognatha_SP7_00000564A_CUBA Cyrtognatha_SP7_00782814_CUBA Cyrtognatha_SP7_00782885_CUBA Cyrtognatha_SP7_00782894_CUBA Cyrtognatha_SP7_00784348_Cuba

Group[ 13 ] n: 6 ;id: Cyrtognatha_SP8_00784418_HSP Cyrtognatha_SP8_00784494_HSP Cyrtognatha_SP8_00784608_HSP Cyrtognatha_SP8_00787174_HSP Cyrtognatha_SP8_00787178_HSP Cyrtognatha_SP8_00787280_HSP

Group[ 14 ] n: 1 ;id: Cyrtognatha_SP10B_00001680A_LA

Group[ 15 ] n: 1 ;id: Tetragnatha_elongata_00001397A_SEUS

*Poisson Tree Processes (PTP), maximum likelihood partition results:*

Species 1 (support = 1.000)

'Tetragnatha_elongata_00001397A_SEUS'

Species 2 (support = 0.925) 'Cyrtognatha_SP1_00001272A_LA','Cyrtognatha_SP1_00001319A_LA','Cyrtognatha_SP1_00001244A_LA','Cyrtognatha_SP1_00001314A_LA','Cyrtognatha_SP1_00001325A_LA'

Species 3 (support = 0.541)

'Cyrtognatha_sp._GB_GU129629','Cyrtognatha_sp._GB_GU129630'

Species 4 (support = 0.142) 'Cyrtognatha_SP7_00782894_CUBA','Cyrtognatha_SP7_00784348_Cuba','Cyrtognatha_SP7_00782885_CUBA','Cyrtognatha_SP7_00782814_CUBA','Cyrtognatha_SP7_00000564A_CUBA','Cyrtognatha_SP7_00000317A_CUBA','Cyrtognatha_SP7_00000232A_CUBA'

Species 5 (support = 1.000)

'Cyrtognatha_SP10B_00001680A_LA'

Species 6 (support = 0.258) 'Cyrtognatha_SP10_00001639A_LA','Cyrtognatha_SP10_00001673A_LA','Cyrtognatha_SP10_00001688A_LA','Cyrtognatha_SP10_00001792A_LA'

Species 7 (support = 0.489)

'Cyrtognatha_SP6_00003173A_JAM','Cyrtognatha_SP6_00003815A_JAM'

Species 8 (support = 0.427) 'Cyrtognatha_SP4_00004444A_JAM','Cyrtognatha_SP4_00004456A_JAM','Cyrtognatha_SP4_00004432A_JAM','Cyrtognatha_SP4_00004209A_JAM','Cyrtognatha_SP4_00004136A_JAM','Cyrtognatha_SP4_00004010A_JAM','Cyrtognatha_SP4_00004462A_JAM','Cyrtognatha_SP4_00002399A_JAM','Cyrtognatha_SP4_00003822A_JAM','Cyrtognatha_SP4_00004029A_JAM','Cyrtognatha_SP4_00004294A_JAM','Cyrtognatha_SP4_00004492A_JAM','Cyrtognatha_SP4_00004206A_JAM','Cyrtognatha_SP4_00003042A_JAM','Cyrtognatha_SP4_00003825A_JAM','Cyrtognatha_SP4_00004170A_JAM','Cyrtognatha_SP4_00004310A_JAM','Cyrtognatha_SP4_00004202A_JAM','Cyrtognatha_SP4_00004335A_JAM','Cyrtognatha_SP4_00004384A_JAM','Cyrtognatha_SP4_00002436A_JAM','Cyrtognatha_SP4_00002589A_JAM','Cyrtognatha_SP4_00002990A_JAM','Cyrtognatha_SP4_00003025A_JAM','Cyrtognatha_SP4_00004283A_JAM','Cyrtognatha_SP4_00004313A_JAM','Cyrtognatha_SP4_00004414A_JAM','Cyrtognatha_SP4_00004474A_JAM'

Species 9 (support = 0.887)

'Cyrtognatha_SP5_00002964A_JAM','Cyrtognatha_SP5_00003183A_JAM'

Species 10 (support = 0.707) 'Cyrtognatha_SP8_00787174_HSP','Cyrtognatha_SP8_00787280_HSP','Cyrtognatha_SP8_00784494_HSP','Cyrtognatha_SP8_00784608_HSP','Cyrtognatha_SP8_00784418_HSP','Cyrtognatha_SP8_00787178_HSP'

Species 11 (support = 0.964) 'Cyrtognatha_elyunquensis_00782116_PR','Cyrtognatha_elyunquensis_00782105_PR','Cyrtognatha_elyunquensis_00782110_PR','Cyrtognatha_elyunquensis_00392843_PR','Cyrtognatha_elyunquensis_00392865_PR','Cyrtognatha_elyunquensis_00392808_PR','Cyrtognatha_elyunquensis_00392732_PR','Cyrtognatha_elyunquensis_00392764_PR','Cyrtognatha_elyunquensis_00392745_PR','Cyrtognatha_elyunquensis_00392763_PR','Cyrtognatha_elyunquensis_00392813_PR','Cyrtognatha_elyunquensis_00392845_PR','Cyrtognatha_elyunquensis_00392853_PR','Cyrtognatha_elyunquensis_00392873_PR','Cyrtognatha_elyunquensis_00392894_PR','Cyrtognatha_elyunquensis_00392911_PR'

Species 12 (support = 1.000)

'Cyrtognatha_SP12_00787269_HSP'

Species 13 (support = 0.818) 'Cyrtognatha_espanola_00782517_HSP','Cyrtognatha_espanola_00782543_HSP','Cyrtognatha_espanola_00787181_HSP','Cyrtognatha_espanola_00784482_HSP','Cyrtognatha_espanola_00785697_HSP','Cyrtognatha_espanola_00787109_HSP','Cyrtognatha_espanola_00787194_HSP','Cyrtognatha_espanola_00785592_HSP','Cyrtognatha_espanola_00782558_HSP','Cyrtognatha_espanola_00782511_HSP','Cyrtognatha_espanola_00785647_HSP','Cyrtognatha_espanola_00785737_HSP','Cyrtognatha_espanola_00787211_HSP','Cyrtognatha_espanola_00782473_HSP','Cyrtognatha_espanola_00782495_HSP','Cyrtognatha_espanola_00782505_HSP','Cyrtognatha_espanola_00782583_HSP','Cyrtognatha_espanola_00782595_HSP','Cyrtognatha_espanola_00784739_HSP','Cyrtognatha_espanola_00784817_HSP','Cyrtognatha_espanola_00785433_HSP','Cyrtognatha_espanola_00785514_HSP','Cyrtognatha_espanola_00785705_HSP','Cyrtognatha_espanola_00785757_HSP'

Species 14 (support = 0.592) 'Cyrtognatha_SP2_00787032_HSP','Cyrtognatha_SP2_00787053_HSP','Cyrtognatha_SP2_00787040_HSP','Cyrtognatha_SP2_00787095_HSP','Cyrtognatha_SP2_00787050_HSP'

Species 15 (support = 0.952)

'Cyrtognatha_SP2B_00786963_HSP'

Species 16 (support = 0.883)

'Cyrtognatha_sp._GB_KY017951','Cyrtognatha_SP2C_00784541_HSP'

**Supplementary Note S2**

***Stepping Stone analyses***

Stepping stone analyses were performed with Path Sampler from MODEL_SELECTION_1.4.1 expansion in BEAST2. We compared a) strict vs relaxed molecular clock and b) relaxed clock + Yule tree prior vs relaxed clock + Birth-Death tree prior. We set the chain length to 1 000 000 while we were increasing the number of steps in analysis from 8 (default) to 100, when we noticed a stabilization in marginal likelihood estimations, a signal of a sufficient number of steps. We left the other settings at their default values.

*Log marginal likelihood estimation for* ***strict clock***

Step theta likelihood contribution ESS

0 1 -3166,512 -105,3758 212,9352

1 0,9667 -3167,36 -102,9227 227,5444

2 0,9342 -3168,0292 -100,4995 132,2904

3 0,9025 -3169,5659 -98,1354 69,4925

4 0,8716 -3168,2059 -95,713 48,6148

5 0,8413 -3169,4491 -93,4025 131,7054

6 0,8119 -3170,8727 -91,129 81,5839

7 0,7831 -3172,5131 -88,8943 73,3964

8 0,7551 -3173,3242 -86,6664 63,7905

9 0,7278 -3172,6508 -84,4306 107,9871

10 0,7012 -3173,3264 -82,2634 136,7318

11 0,6753 -3174,7972 -80,1478 184,0397

12 0,6501 -3176,3646 -78,068 154,7375

13 0,6255 -3177,1735 -75,9978 83,4894

14 0,6016 -3176,0643 -73,9127 148,8682

15 0,5783 -3178,7673 -71,949 198,799

16 0,5557 -3179,0164 -69,9611 196,0003

17 0,5337 -3179,7433 -68,0146 213,2429

18 0,5123 -3181,3269 -66,1163 94,5982

19 0,4915 -3183,4717 -64,2618 65,8536

20 0,4713 -3183,7256 -62,3967 139,9312

21 0,4517 -3186,4156 -60,6121 138,9925

22 0,4327 -3187,1184 -58,8136 108,706

23 0,4142 -3189,1798 -57,0735 91,2567

24 0,3964 -3189,3818 -55,3291 208,1114

25 0,379 -3193,0819 -53,6774 105,1145

26 0,3622 -3192,3344 -51,9733 139,7569

27 0,3459 -3198,9239 -50,4231 126,8411

28 0,3302 -3200,3669 -48,8167 128,0579

29 0,3149 -3201,7995 -47,2412 45,62

30 0,3002 -3205,1222 -45,7188 56,3314

31 0,2859 -3205,1216 -44,1752 97,0893

32 0,2721 -3211,1566 -42,7461 130,4207

33 0,2588 -3211,3268 -41,2639 26,6337

34 0,246 -3214,0342 -39,8427 162,7

35 0,2336 -3219,1064 -38,4792 103,8924

36 0,2217 -3223,4382 -37,1354 232,0475

37 0,2101 -3230,1266 -35,8377 137,6251

38 0,1991 -3234,629 -34,5506 73,509

39 0,1884 -3237,93 -33,2616 126,2802

40 0,1781 -3242,5484 -32,0167 106,6727

41 0,1683 -3249,1978 -30,8213 157,4515

42 0,1588 -3250,8671 -29,5987 82,9722

43 0,1497 -3260,948 -28,4842 106,7139

44 0,141 -3265,9652 -27,3451 213,1339

45 0,1326 -3272,9189 -26,246 248,741

46 0,1246 -3280,0239 -25,1667 232,8823

47 0,1169 -3291,1158 -24,1508 349,1787

48 0,1096 -3300,9565 -23,1449 226,4208

49 0,1026 -3309,3658 -22,1455 116,3011

50 0,0959 -3322,1666 -21,2037 189,5092

51 0,0895 -3328,4448 -20,2322 253,0974

52 0,0835 -3347,2986 -19,3696 100,9421

53 0,0777 -3360,8013 -18,4906 91,8557

54 0,0722 -3374,0441 -17,6208 131,9619

55 0,067 -3389,2448 -16,7896 291,7689

56 0,0621 -3400,7277 -15,9637 146,0406

57 0,0574 -3420,6576 -15,1869 209,6601

58 0,0529 -3467,0084 -14,5561 18,1222

59 0,0488 -3457,4091 -13,6833 115,1136

60 0,0448 -3546,0773 -13,2402 58,5185

61 0,0411 -3503,0711 -12,2858 103,7215

62 0,0376 -3529,1886 -11,6229 112,1372

63 0,0343 -3558,3169 -10,9866 84,1308

64 0,0312 -3612,7123 -10,4403 27,0929

65 0,0284 -3629,9195 -9,8035 24,155

66 0,0257 -3666,8096 -9,2209 107,7254

67 0,0232 -3696,7848 -8,6422 45,6003

68 0,0208 -3698,8651 -8,023 23,5313

69 0,0187 -3776,6417 -7,582 65,2599

70 0,0167 -3790,9917 -7,0187 142,2954

71 0,0149 -3833,6086 -6,5331 81,4664

72 0,0132 -3865,3952 -6,0463 30,5431

73 0,0116 -3925,9241 -5,6159 128,667

74 0,0102 -3961,5824 -5,1606 199,0962

75 0,0089 -4014,4043 -4,7475 109,7371

76 0,0077 -4094,3384 -4,3794 177,3572

77 0,0066 -4154,3186 -4,0035 196,7489

78 0,0057 -4228,3746 -3,6488 120,4398

79 0,0048 -4286,6393 -3,2969 150,1925

80 0,0041 -4498,2801 -3,0852 97,4459

81 0,0034 -4482,2054 -2,6814 120,5365

82 0,0028 -5004,7497 -2,6563 85,2674

83 0,0023 -6221,121 -3,1755 6,8405

84 0,0019 -5969,5525 -2,5119 8,9519

85 0,0015 -25547,8556 -8,2792 365,035

86 0,0012 -25784,6086 -6,9873 354,88

87 0,0009 -26190,4 -5,8504 439,3748

88 0,0007 -26598,0998 -4,8198 327,5251

89 0,0005 -26853,2871 -3,8484 436,8897

90 0,0003 -27537,8804 -3,0521 501

91 0,0002 -28059,1244 -2,3258 474,9046

92 0,0001 -28813,3087 -1,7111 501

93 0,0001 -29339,6248 -1,1795 462,6986

94 0 -30133,5573 -0,7584 501

95 0 -30539,9724 -0,4284 344,5144

96 0 -30523,8619 -0,1967 293,7826

97 0 -30726,9551 -0,0622 405,4243

98 0 -30855,6419 -0,0069 401,6161

99 0 -30734,2437 0 428,4459

sum(ESS) = 16938,7086

**marginal L estimate = -3265.360447254418 (strict clock, yule tree prior)**

__________________________________________________________________

*Log marginal likelihood estimation for* ***relaxed clock***

Step theta likelihood contribution ESS

0 1 -3166,592 -105,3793 75,3206

1 0,9667 -3167,8821 -102,9419 133,5716

2 0,9342 -3167,8703 -100,4945 118,7878

3 0,9025 -3168,8038 -98,1115 135,0128

4 0,8716 -3169,7881 -95,7607 124,2002

5 0,8413 -3170,3459 -93,4291 165,6454

6 0,8119 -3170,0848 -91,1066 130,2024

7 0,7831 -3172,126 -88,8822 159,8446

8 0,7551 -3171,6823 -86,6208 288,7492

9 0,7278 -3173,2406 -84,4461 146,6647

10 0,7012 -3173,8944 -82,2792 116,8048

11 0,6753 -3175,4177 -80,1641 81,7196

12 0,6501 -3175,3908 -78,0436 136,0939

13 0,6255 -3175,9949 -75,9693 99,5361

14 0,6016 -3176,6617 -73,9255 185,2926

15 0,5783 -3179,1553 -71,961 94,6827

16 0,5557 -3180,3951 -69,993 113,1611

17 0,5337 -3181,0533 -68,0456 76,6008

18 0,5123 -3181,9405 -66,1334 108,6478

19 0,4915 -3181,87 -64,2279 240,9708

20 0,4713 -3182,7867 -62,3807 111,5118

21 0,4517 -3185,1482 -60,5865 84,618

22 0,4327 -3187,0495 -58,8139 66,5751

23 0,4142 -3189,0004 -57,071 90,4977

24 0,3964 -3192,967 -55,3948 21,8718

25 0,379 -3192,3032 -53,6613 119,0703

26 0,3622 -3193,8907 -52,0008 173,6714

27 0,3459 -3195,8704 -50,3759 85,1265

28 0,3302 -3199,2351 -48,8015 37,5379

29 0,3149 -3199,3361 -47,2027 198,2303

30 0,3002 -3202,3969 -45,6777 79,3795

31 0,2859 -3206,4023 -44,1934 130,8049

32 0,2721 -3209,2834 -42,723 55,9586

33 0,2588 -3213,4081 -41,2895 51,4452

34 0,246 -3217,0506 -39,8801 119,1706

35 0,2336 -3220,7936 -38,4997 126,781

36 0,2217 -3224,5574 -37,1449 104,7639

37 0,2101 -3229,1117 -35,8248 211,1973

38 0,1991 -3235,0345 -34,5465 227,4253

39 0,1884 -3236,6451 -33,2471 76,9713

40 0,1781 -3240,8632 -31,9969 183,9931

41 0,1683 -3248,8365 -30,8163 205,1496

42 0,1588 -3250,0378 -29,5958 74,1073

43 0,1497 -3260,8355 -28,478 239,7116

44 0,141 -3263,3348 -27,3202 90,3927

45 0,1326 -3276,746 -26,2835 188,7623

46 0,1246 -3281,2681 -25,1807 111,1635

47 0,1169 -3286,4848 -24,1169 207,4677

48 0,1096 -3300,5421 -23,1422 171,4449

49 0,1026 -3307,2032 -22,1368 85,4808

50 0,0959 -3325,5447 -21,2194 127,3793

51 0,0895 -3329,3952 -20,2417 217,807

52 0,0835 -3342,6008 -19,3428 212,4648

53 0,0777 -3348,3407 -18,4137 290,1643

54 0,0722 -3383,2761 -17,6685 75,1244

55 0,067 -3391,5301 -16,7985 337,3632

56 0,0621 -3412,6443 -16,0149 81,8804

57 0,0574 -3421,0322 -15,185 174,149

58 0,0529 -3444,6459 -14,4568 60,4691

59 0,0488 -3458,0497 -13,683 110,7889

60 0,0448 -3487,1214 -13,0119 63,4397

61 0,0411 -3507,7251 -12,3006 71,0564

62 0,0376 -3568,793 -11,7646 28,5381

63 0,0343 -3596,4268 -11,1115 13,4034

64 0,0312 -3585,1778 -10,3653 34,9414

65 0,0284 -3602,2998 -9,717 89,3981

66 0,0257 -3649,3439 -9,1865 45,5132

67 0,0232 -3713,8765 -8,6831 36,6658

68 0,0208 -3747,3082 -8,134 71,3098

69 0,0187 -3785,3366 -7,594 46,7379

70 0,0167 -3805,2188 -7,0521 180,8024

71 0,0149 -3857,1471 -6,5748 343,7351

72 0,0132 -3928,4296 -6,1442 228,3646

73 0,0116 -3955,6565 -5,6559 287,5432

74 0,0102 -3992,2802 -5,2078 133,8398

75 0,0089 -4049,2691 -4,7942 170,5619

76 0,0077 -4091,5925 -4,3744 203,9364

77 0,0066 -4172,5717 -4,0194 251,0644

78 0,0057 -4268,7821 -3,6896 133,7595

79 0,0048 -4380,1467 -3,3756 166,3033

80 0,0041 -4465,999 -3,0743 111,7059

81 0,0034 -4698,9119 -2,8516 114,1356

82 0,0028 -4893,6696 -2,6277 31,3143

83 0,0023 -5288,6005 -2,4413 33,3799

84 0,0019 -6301,765 -2,6053 13,7985

85 0,0015 -5950,5448 -2,0442 10,6977

86 0,0012 -8133,5008 -2,2817 10,8297

87 0,0009 -8100,6126 -1,8781 15,0411

88 0,0007 -9069,5025 -1,65 47,1254

89 0,0005 -9446,2906 -1,3589 29,5075

90 0,0003 -9858,3391 -1,087 144,3416

91 0,0002 -9951,0825 -0,8198 46,915

92 0,0001 -9825,8994 -0,5796 79,6345

93 0,0001 -10006,8046 -0,3996 71,0567

94 0 -10053,351 -0,2517 129,2332

95 0 -9813,3769 -0,1371 63,1248

96 0 -9767,4613 -0,0628 36,9453

97 0 -10058,9598 -0,0203 142,6042

98 0 -10617,4967 -0,0024 19,9389

99 0 -10831,9274 0 8,4632

sum(ESS) = 11910,0893

**marginal L estimate = -3238.2528045221197 (relaxed clock, yule tree prior)**

__________________________________________________________________

*Log marginal likelihood estimation for relaxed clock with* ***Birth-Death tree prior****.*

Step theta likelihood contribution ESS

0 1 -3167,8662 -105,4174 194,8028

1 0,9667 -3169,4076 -102,9891 84,2441

2 0,9342 -3169,4298 -100,5426 122,5461

3 0,9025 -3169,5619 -98,1323 147,9107

4 0,8716 -3170,4999 -95,7799 155,0177

5 0,8413 -3170,8032 -93,4403 117,751

6 0,8119 -3171,9935 -91,1615 142,347

7 0,7831 -3171,2578 -88,8559 93,5559

8 0,7551 -3172,1979 -86,6337 105,6951

9 0,7278 -3173,4393 -84,4496 156,1453

10 0,7012 -3174,2979 -82,2892 156,6703

11 0,6753 -3174,2759 -80,1331 197,4284

12 0,6501 -3175,3269 -78,0417 95,9852

13 0,6255 -3176,5535 -75,9803 127,4343

14 0,6016 -3176,5042 -73,9226 423,5654

15 0,5783 -3177,6923 -71,9263 93,914

16 0,5557 -3178,6486 -69,9533 196,0384

17 0,5337 -3181,1239 -68,0446 49,2389

18 0,5123 -3182,1938 -66,1364 241,3194

19 0,4915 -3181,2359 -64,2138 206,8736

20 0,4713 -3183,7835 -62,3962 197,3433

21 0,4517 -3184,7493 -60,5782 112,1403

22 0,4327 -3186,9609 -58,8112 81,5142

23 0,4142 -3189,7367 -57,0849 60,1298

24 0,3964 -3191,4828 -55,3656 132,0394

25 0,379 -3192,7222 -53,6694 83,119

26 0,3622 -3193,8432 -51,9996 66,4822

27 0,3459 -3196,645 -50,389 93,3592

28 0,3302 -3198,4116 -48,7884 160,6657

29 0,3149 -3200,7029 -47,2209 190,354

30 0,3002 -3200,7291 -45,6537 106,4118

31 0,2859 -3207,5679 -44,2089 109,1808

32 0,2721 -3208,036 -42,706 147,2511

33 0,2588 -3211,8061 -41,2686 97,5815

34 0,246 -3215,0749 -39,8614 53,2055

35 0,2336 -3218,0663 -38,4696 111,1175

36 0,2217 -3223,0931 -37,1304 112,0944

37 0,2101 -3227,841 -35,8129 154,6074

38 0,1991 -3227,0611 -34,4578 80,4919

39 0,1884 -3235,2688 -33,2317 72,2192

40 0,1781 -3240,6915 -31,9967 218,9329

41 0,1683 -3244,1718 -30,7705 345,0764

42 0,1588 -3251,8779 -29,613 43,1846

43 0,1497 -3252,7402 -28,4103 102,1561

44 0,141 -3265,4591 -27,3421 223,0962

45 0,1326 -3271,0038 -26,2287 145,6628

46 0,1246 -3289,7384 -25,2713 19,8663

47 0,1169 -3286,57 -24,1167 318,566

48 0,1096 -3296,9107 -23,1138 203,8501

49 0,1026 -3312,1038 -22,1654 369,5284

50 0,0959 -3316,8538 -21,1656 204,8231

51 0,0895 -3328,8932 -20,238 194,461

52 0,0835 -3341,422 -19,3307 264,1847

53 0,0777 -3353,8413 -18,454 222,111

54 0,0722 -3370,0218 -17,6013 225,0183

55 0,067 -3388,4011 -16,7899 109,5278

56 0,0621 -3403,4816 -15,9813 123,9512

57 0,0574 -3417,9926 -15,1733 123,6978

58 0,0529 -3429,9066 -14,3887 125,3195

59 0,0488 -3462,4792 -13,7105 69,3568

60 0,0448 -3468,4213 -12,9284 169,9038

61 0,0411 -3520,6359 -12,3585 19,3632

62 0,0376 -3536,5695 -11,6506 16,0674

63 0,0343 -3540,5176 -10,9326 54,1153

64 0,0312 -3574,4473 -10,3258 97,6817

65 0,0284 -3611,354 -9,7462 48,4532

66 0,0257 -3641,9533 -9,1589 65,8081

67 0,0232 -3698,4103 -8,6396 36,6332

68 0,0208 -3734,0655 -8,0968 45,8815

69 0,0187 -3793,2692 -7,6069 213,9412

70 0,0167 -3761,046 -6,9597 62,4673

71 0,0149 -3820,242 -6,5092 161,6952

72 0,0132 -3858,5886 -6,0276 209,1842

73 0,0116 -3917,92 -5,6048 159,1128

74 0,0102 -3964,168 -5,1671 231,0577

75 0,0089 -3993,0607 -4,7235 174,0112

76 0,0077 -4100,4654 -4,3905 167,3533

77 0,0066 -4146,744 -3,9991 214,4317

78 0,0057 -4253,618 -3,6845 165,7257

79 0,0048 -4315,7386 -3,3228 188,5687

80 0,0041 -4392,7015 -3,0009 136,6995

81 0,0034 -4639,5018 -2,8374 50,7792

82 0,0028 -4783,8317 -2,538 70,1195

83 0,0023 -6242,7237 -3,1015 16,2908

84 0,0019 -5839,8144 -2,3651 26,944

85 0,0015 -9460,3795 -3,7327 10,1595

86 0,0012 -25797,5868 -6,9927 319,9602

87 0,0009 -26174,3675 -5,843 345,6093

88 0,0007 -26632,659 -4,8197 405,777

89 0,0005 -26891,3724 -3,8532 501

90 0,0003 -27399,2205 -3,0336 482,6985

91 0,0002 -28057,9264 -2,3222 316,7502

92 0,0001 -28671,4335 -1,7008 501

93 0,0001 -29466,3248 -1,1845 369,2696

94 0 -29849,6807 -0,7501 421,8298

95 0 -30520,699 -0,4281 501

96 0 -30783,7763 -0,1984 458,2701

97 0 -30678,1945 -0,0621 362,4901

98 0 -30880,6715 -0,0069 501

99 0 -30533,3598 0 486,8265

sum(ESS) = 17768,093

**marginal L estimate = -3259.613809934745 (relaxed clock, Birth-Death tree prior)**

__________________________________________________________________

***Likelihood ratio test (LRT) and Bayes factor (BF)***

LRT test was performed with lr.test in R package extRemes.

Log BF was calculated as (log marginal likelihood of model 1 – log marginal likelihood of model 2).

(https://revbayes.github.io/tutorials/model_selection_bayes_factors/bf_intro.html)

**Strict vs relaxed clock: LRT = 54.2 (p < 0.001); logBF = 27.1 in favor of relaxed clock**

**Yule tree vs Birth-Death tree: LRT = 42.7 (p < 0.001); logBF = 21.4 in favor of Yule tree prior**
